# Is the *GSTM1* null polymorphism a risk factor in Primary Open Angle Glaucoma?

**Published:** 2011-06-23

**Authors:** Auta Viviane Rocha, Teddy Talbot, Thiago Magalhães da Silva, Maria Clarinda Almeida, Carlos Alberto Menezes, Giuliano Di Pietro, Fabrício Rios-Santos

**Affiliations:** 1 Universidade Estadual de Santa Cruz, Departamento de Ciências da Saúde, Laboratório de Farmacogenômica e Epidemiologia Molecular (LAFEM), Ilhéus, Bahia, Brazil; 2 Universidade Federal de Sergipe - Campus de Lagarto, Laboratório de Genética, São Cristovão, Sergipe, Brazil

## Abstract

**Purpose:**

To investigate the association of glutathione S-transferase (*GST*) *GSTM1*, *GSTT1*, and *GSTP1* genes with the risk of primary open angle glaucoma (POAG) and clinical features of the disease.

**Methods:**

We conducted a case-control study that included 87 Brazilian patients with POAG and 85 healthy controls matched for age, ethnicity, and sex, whose blood samples were genotyped for polymorphisms in *GST* genes using polymerase chain reaction (PCR) based methods.

**Results:**

The *GSTM1* null polymorphism was significantly more common in the POAG than in the controls group (OR: 2.1, 95% CI: 1.13–3.9; p=0.018). The combined *GSTM1* null/*GSTT1*+ genotype and *GSTM1* null/*GSTP1* Ile/Val or Val/Val was more prevalent in POAG patients, being a risk factor for POAG (OR: 2.4, 95% CI: 1.16–4.9; p=0.016 and OR: 2.7, 95% CI: 1.07–6.74; p=0.033, respectively). The *GSTM1* null/*GSTT1*+ genotype were associated with higher levels of IOP of both eyes and with more severe defect of the right eye optic nerve. The *GSTM1* null/*GSTP1* Ile/Val or Val/Val genotypes were associated with higher levels of IOP and more advanced defect of the right eye optic nerve and visual field.

**Conclusions:**

We demonstrate that *GSTM1* null polymorphism is associated with POAG in the Brazilian population.

## Introduction

Glaucoma is a progressive neuropathy which has a characteristic pattern of optic nerve and visual field damage. Primary open angle glaucoma (POAG) is the most common form of glaucoma affecting 2% of the world’s population over 40 year’s old [1]. It is considered the second cause of irreversible blindness worldwide. Quigley et al. [2] estimate that 79.6 million people will be affected with glaucoma by 2020.

Although the pathophysiology is poorly understood, it is believed that environmental and genetic factors may play an important role in POAG. The increase of intra ocular pressure (IOP) is known to be one of the major risk factors for the disease. POAG exhibits a heritable susceptibility consistent with a complex trait inheritance and only three genes have been implicated in the pathogenesis of the disease, myocilin (*MYOC*) [3], optineurin (*OPTN*) [3], and WD repeat-containing protein 36 (*WDR36*) [3]. Mutations in *MYOC* account for 1%–4% of POAG depending on the population studied [3]. Other studies suggest that oxidative stress may be involved in the pathophysiology of POAG [4]. Chronic oxidative stress may contribute to increase of intraocular pressure by increased resistance of aqueous humor outflow through trabecular meshwork (TM).

Glutathione S-transferases (GST) are polymorphic enzymes that catalyze the neutralization of free radicals by their conjugation with glutathione and thus render the products more water-soluble [5]. Several studies have demonstrated that *GST* genetic polymorphisms are associated with a higher risk of developing POAG in different populations [6,7]. However, these results were not confirmed in other studies [8]. Thus, the aim of the present study was to characterize the southern Bahia, Brazil population for *GSTM1*, *GSTT1*, and *GSTP1* polymorphisms and determine the relative risk of POAG associated with these polymorphisms.

## Methods

### Patients and controls

Eighty seven patients with POAG (27 men and 60 women; mean age 63.5±11.02) were recruited from out-patient clinic and a group of 85 healthy controls (29 men and 56 women; mean age 61.8±11.23) originating from public awareness programs on glaucoma donors and blood banks were selected for the study. All individuals live in the south of Bahia State, Brazil. Information concerning dietary habits, history of disease and lifestyle (including tobacco smoking) was obtained from a socioeconomic questionnaire administered to both groups. All volunteers signed a consent form approved by the local ethics committee of the Universidade Estadual de Santa Cruz (UESC).

### Clinical evaluation

All patients underwent complete ophthalmologic evaluation that included medical history, best-corrected visual acuity, slit-lamp biomicroscopy with and without dilation, applanation tonometry, dilated fundoscopy, ophthalmoscopy of the optic disk with a 78-diopter lens, gonioscopy, and computerized visual fields. Humphrey automated perimeter strategy C-24–2 visual field was done for glaucoma patients and frequency doubling perimeter (FDT^®^) visual field for the controls. The inclusion criteria for the case group were initial IOP (before treatment) above 21 mmHg, defect suggestive of glaucoma on the optic nerve (ON) and visual field (VF). Cup/Disc ratio was measured according to Anderson's [9] criteria and optic nerve defect was qualitatively analyzed and further defined using standard ranking as mild, moderate or advanced damage according to the severity of the disease (Table 1). To evaluate visual field damage, VF map was analyzed and VF score was assigned according to the Brusini Glaucoma Staging System [10]. Patients with history of surgery, uveitis, trauma or secondary glaucoma were excluded. The inclusion criteria for the control group were IOP below 21 mmHg, the anterior chamber angle open, optic nerve and visual fields without abnormalities suggestive of glaucoma. Patients with malignant disease or autoimmune disease were excluded.

**Table 1:** Classification of defects of the optic nerve damage.

**Defect**	**Type**	**Description**
0	Normal	Without lesion
1	Initial defect	Focal deficiency in 1 or more pole that do not reach up to the papillary edge
2	Small defect	Focal deficiency in 1 pole that reach to the papillary edge
3	Middle Defect	Focal deficiency in 1 or more pole that reach papillary edge (Notch)
4	Middle severe defect	Focal deficiency in 1 or more pole that reach the papillary edge (Notch) plus hemorrhage or β zone
5	Severe defect	Cup/Disc ratio above 09/09
6	Null	Cup/Disc ratio total (10/10)

### Sample collection and DNA analysis

Ten milliliters of peripheral blood was collected in EDTA vacutainer tubes from all participating individuals after obtaining their written consent. Genomic DNA extraction was performed from whole blood using the FlexiGene DNA Kit (Qiagen, Boston, MA). *GSTM1, GSTT1, and GSTP1* polymorphism analyses were performed by PCR [11]. Briefly, for *GSTM1* and *GSTT1* genotyping 50-500 ng of DNA was amplified in a 50-µl multiplex reaction mixture containing 20 pmol of each of the following *GSTM1* primers (G1 - 5'-GAA CTC CCT GAA AAG CTA AAG C-3' and G2 - 5'-GTT GGG CTC AAA TAT ACG GTG G-3'), and of the following *GSTT1* primers (T1 - 5'-TTC CTT ACT GGT CCT CAC ATC TC-3' and T2 - 5'-TCA CCG GAT CAT GGC CAG CA-3'). As an internal control, a fragment of the human b-globin gene was also amplified (GH20 5'-GAA GAG CCA AGG ACA GGT AC-3' and PC04 5'-CAA CTT CAT CCA CGT TCA CC-3') in a medium consisting of 1.5 mM MgCl_2_, 200 µmol dNTPs, 5 µl 10× PCR buffer (10× 500 mM KCl, 100 mM Tris-HCl, pH 9.0), and 2 U TaqDNA polymerase (Promega, Madison, WI). The PCR protocol included an initial melting temperature of 94 ºC (5 min) followed by 35 cycles of amplification (2 min at 94 ºC, 1 min at 59 ºC, and extension for 1 min at 72 ºC). A final 10-min extension step (72 ºC) terminated the process. The final PCR product from co-amplification of *GSTM1* (215 bp) and *GSTT1* (480 bp) was visualized on an ethidium bromide-stained 2.0% agarose gel. The subjects were classified as either positive (when at least one copy of the gene was present) or null genotypes. To genotype the *GSTP1* BsmaI polymorphism the PCR-RFLP method was applied as follows: 50-500 ng of DNA was amplified in a total reaction volume of 25 µl containing 20 mM Tris- HCl, 50 mM KCl, 1.5 mM MgCl_2_, 2 mM of each deoxynucleotide triphosphate, 100 ng/ µl of each primer (P105F 5'-ACC CCA GGG CTC TAT GGG AA-3' and P105R 5'-TGA GGG CAC AAG AAG CCC CT-3'), and 1.25 U AmpliTaq DNA polymerase. PCR was carried out in an Eppendorf Gradient Thermocycler, with 5 min of pretreatment at 95 ºC, 30 cycles of 30 s at 57 ºC, 30 s at 72 ºC, and 30 s at 94 ºC, followed by 5 min at 72 ºC. After amplification, PCR products (20 µl) were cleaved by 5 U BsmaI enzyme at 55 ºC for 2 h and then subjected to electrophoresis on 3.5% agarose gel at 40 V for 2 h and visualized using ethidium bromide. The primers P105F and P105R generated an amplified fragment of 176 bp corresponding to the wild genotype (Ile/Ile). After cleavage with the restriction enzyme BsmaI, individuals with the variant allele (Ile/Val) will show three bands of 176 (Ile), 91, and 85 (Val) bp, respectively. The mutant homozygote (Val/Val) has both alleles cleaved, showing two bands (81 and 91 bp).

### Statistical analysis

Age of patients and control group was compared using the Student *t*-test. χ^2^ test was used to assess differences in allelic and genotype frequencies between groups. Odds ratio (OR) and confidence interval 95% (CI) were used to analyze the risk of POAG associated with *GSTM1, GSTP1*, and *GSTT1* genotypes, alone or in combination. A multivariate logistic regression model was applied to assess the risk of POAG attributed to polymorphisms of *GST*s after adjusting for gender, age, ethnicity and tobacco smoking. The reference group consisted of individuals with three putative low-risk genotypes, i.e., the presence of *GSTM1* (non-deleted), *GSTT1* (non-deleted), and *GSTP1* (homozygous Ile-104) functional alleles. ANOVA (ANOVA) was used to assess the association of *GST*’s genotype combinations with clinical parameters in the cohort of POAG patients. Kolmogorov–Smirnov testing was performed on all data before analysis to determine if data was normally distributed. Non-normally distributed data was analyzed using ANOVA on ranks with the Tukey test employed for post hoc analysis. Mann–Whitney U test was used to assess differences in clinical variables according to *GST*s genotypes. A Hardy–Weinberg equilibrium test was also performed using χ^2^ tests. All tests were performed with Statistical Package for the Social Sciences (SPSS-10, Inc., Chicago, IL).

## Results

The individuals enrolled for the study were matched for age, sex, and ethnicity to obtain two homogeneous groups. The glaucoma group consisted of 87 individuals (27 males and 60 females with the mean age of 63.5±11.02 years old). The control group consisted of 85 individuals (29 males and 56 females with the mean age of 61.8±11.23 years old). There was no statistically significant difference (p>0.05) regarding gender and age between groups. On the other hand, cup/disc ratio and IOP levels were almost two folds higher in the POAG group when compared with the control group (0.8±0.1 and 0.3±0.1; p<0.001, respectively, Table 2).

**Table 2:** Demographic and ophthalmologic data of the individuals studied.

**Variables**	**Glaucoma group n=87**	**Control group n=85**	p value*
**Gender**			
Male	27 (31%)	29 (34..1%)	0.666
Female	60 (69%)	56 (65..9%)	
Age (mean)	63..46 (±11.02)	61.67 (±11.23)	0.323
**Ethnicity**			
White^1^	17 (19.5%)	20 (23.5%)	0.609
Not White	70 (80.5%)	65 (76.5%)	
IOP RE	26.73±3.38	14.28±2.18	p<0.001
IOP LE	26.19±3.93	13.95±1.99	p<0.001
C/D RE	0.8 (0.4–10)	0.3 (0.2–0.4)	p<0.001
C/D LE	0.7 (0.4–10)	0.3 (0.2–0.4)	p<0.001

^1^According to each individual self-declared ethnicity (IBGE); IOP=intra ocular pressure in mmHg; RE: right eye; LE: left eye; C/D: cup disc/ratio. *Student *t*-test.

The *GSTM1* null genotype frequency was significantly higher in POAG patients (49.4%) than in controls (31.8%). The *GSTM1* null genotype had an increased risk of developing POAG (OR=2.01; 95% CI: 1.13–3.9; p=0.018). The *GSTT1* null genotype was more frequent in the control group (28.2%) than in the case group (17.2%). However, such difference was not statistically significant (OR: 0.53; 95% CI: 0.25–1.01; p=0.085). The *GSTP1* genotype frequency did not differ between the two groups (Table 3).

**Table 3:** Risk of POAG between GST genotypes.

**Genotype**	**Cases n (%)**	**Control n (%)**	**OR (IC 95)**	p value*
***GSTM1***				
*GSTM1*+	44 (50.6)	58 (68.2)	Ref.	־
*GSTM1*-	43 (49.4)	27 (31.8)	2.1 (1.13–3.9)	0.018
***GSTT1***				
*GSTT1*+	72 (82.8)	61 (71.8)	Ref.	־
*GSTT1*-	15 (17.2)	24 (28.2)	0.53 (0.25–1.01)	0.085
***GSTP1***				
Ile/Ile	44 (50.6)	45 (52.9)	Ref.	־
Ile/Val	35 (40.2)	34 (40)	1.05 (0.56–2.0)	0.873
Val/Val	08 (09.2)	06 (07.1)	1.36 (0.44–4.25)	0.592
Ile/Val or Val/Val	43 (49.4)	40 (47.1)	1.01 (0.6–2.0)	0.756
Ile	123 (71.0)	124 (73.0)	Ref.	־
Val	51 (39.0)	46 (27.0)	1.12 (0.7–1.8)	0.643

Reference groups: *GSTM1+* (non-deleted), *GSTT1*+ (non-deleted) and *GSTP1* wild-type allele. *χ^2^ test.

The association between *GSTM1* and POAG remained statistically significant when adjusted for gender, age, ethnicity, tobacco smoking and *GST*s genotypes by multivariate logistic regression model (OR=2.22; 95% CI: 1.14–4.31; p=0.018; data not shown).

Table 4 shows the frequency of combined genotypes. The *GSTM1* null/*GSTT1*+ was significantly higher in POAG patients than in controls (OR=2.4; 95% IC: 1.16–4.9; p=0.016) and the association of genotypes *GSMT1* null and *GSTP1* Ile/Val or Val/Val was also significant (OR=2.7; 95% CI: 1.07–6.74; p=0.033). The difference in frequency of the *GSTM* null/*GSTT* null genotype was not significant (OR=0.93; 95% CI: 0.31–2.74; p=0.894).

**Table 4:** Distribution of *GST* genotypes combined between cases and controls.

Genotype combinations	Groups			
** **	**Cases n (%)**	**Controls n (%)**	**OR (IC 95)**	p value*
***GSTM1*/*GSTT1***				
M1+/T1+	36 (41.4)	43 (50.6)	Ref.	־
M1+/T1-	8 (9.2)	15 (17.6)	0.64 (0.24–1.67)	0.358
M1-/T1+	36 (41.4)	18 (21.2)	2.4 (1.16–4.9)	0.016
M1-/T1-	7 (8)	9 (10.6)	0.93 (0.31–2.74)	0.894
***GSTM1*/*GSTP1***				
M1+/Ile/Ile	24 (27.6)	28 (32.9)	Ref.	־
M1+/ Ile/Val or Val/Val	20 (23.0)	30 (35.3)	0.78 (0.35–1.7)	0.53
M1-/ Ile/Val or Val/Val	23 (26.4)	10 (11.8)	2.7 (1.07–6.74)	0.033
M1-/Ile/Ile	20 (23.0)	17 (20)	1.37 (0.6–3.2)	0.463
***GSTT1*/*GSTP1***				
T1+/Ile/Ile	35 (40.2)	34 (40.0)	Ref.	־
T1+/ Ile/Val or Val/Val	37 (42.5)	27 (31.8)	1.33 (0.67–2.64)	0.412
T1-/ Ile/Val or Val/Val	6 (6.9)	13 (15.3)	0.45 (0.15–1.3)	0.138
T1-/Ile/Ile	9 (10.3)	11 (12.9)	0.8 (0.29–2.16)	0.652

Reference groups: *GSTM1+* (non-deleted), *GSTT1*+ (non-deleted) and *GSTP1* wild-type allele. *χ^2^ test.

Table 5 shows the clinical features associated with the *GSTM1* genotype in the POAG group by applying the nonparametric Mann–Whitney test. The results show that the *GSTM1* null genotypes were associated with higher IOP values in right and left eyes (p=0.009 and 0.035, respectively). Other results show that the *GSTM1* null genotypes are associated with severe optic nerve damage (grade 4; p=0.006). The differences were not statistically significant (data not shown) for the *GSTP1* and *GSTT1* genotypes.

**Table 5:** Mean values of clinical parameters evaluated for the case group according to the *GSTM1* genotypes.

**Variables**	**Genotype**	**n**	**Mean**	**SD**	p value*
IOPRb	*GSTM1*-	34	24.44	5.58	0.009
	*GSTM1*+	37	21	5.06	
IOPLb	*GSTM1*-	34	23.65	6.24	0.035
	*GSTM1*+	37	20.73	6.26	
ONR: DEF	*GSTM1*-	39	2.54	2.11	0.006
	*GSTM1*+	39	1.33	1.64	
ONL: DEF	*GSTM1*-	39	2.21	2.1	0.186
	*GSTM1*+	39	1.56	1.9	
VFMDR	*GSTM1*-	37	−8.73	8.0	0.239
	*GSTM1*+	33	−6.04	6.0	
VFPSDR	*GSTM1*-	37	5.48	3.76	0.760
	*GSTM1*+	33	5.61	3.09	
VFDEFR	*GSTM*1-	38	3.11	2.31	0.033
	*GSTM1*+	33	1.94	1.73	
VFMDL	*GSTM1*-	37	−8.67	8.8	0.181
	*GSTM1*+	31	−5.47	5.6	
VFPSDL	*GSTM1*-	37	4.80	2.92	0.486
	*GSTM1*+	31	5.46	2.95	
VFDEFL	*GSTM1*-	38	2.79	2.27	0.279
	*GSTM1*+	31	2.1	1.87	

*Mann–Whitney test. IOPRb: Intra ocular pressure of right eye before treatment; IOPLb: Intra ocular pressure of left eye before treatment; ONR:DEF: right eye optic nerve defect; OotherL:DEF: left eye optic nerve defect; VFMDR: visual field mean deviation right eye; VFPSDR: visual field pattern standard deviation right eye; VFDEFR: visual field defect of right eye; VFMDL: visual field mean deviation of left eye; VFPSDL: visual field pattern standard deviation left eye; VFDEFL: visual field defect of left eye.

**Figure 1:**
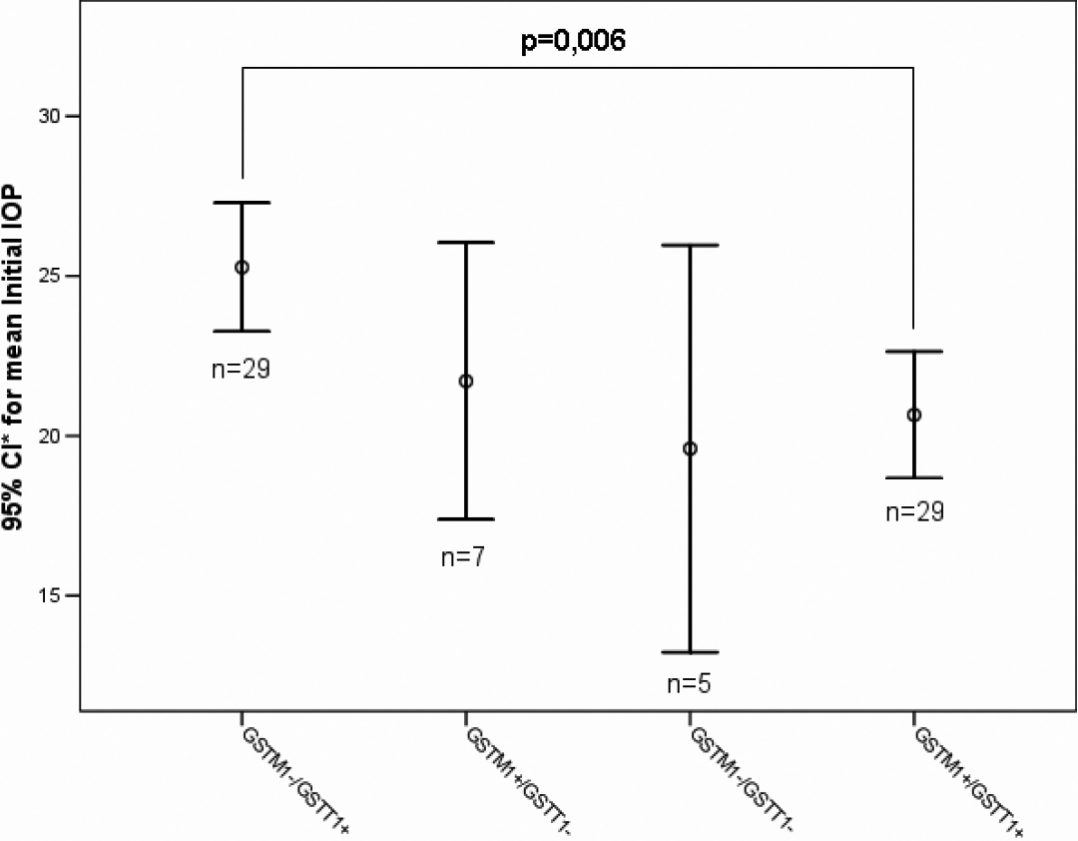
Differences of means for the parameter initial IOP of the right eye for the combined genotypes of *GSTM1* and *GSTT1.* The mean of initial IOP to the right eye was significantly higher in the POAG patients carrying the GSTM1-/GSTT1+ genotype combination compared to those with the GSTM1+/GSTT1+ combination. *CI: Confidence Interval.

**Figure 2:**
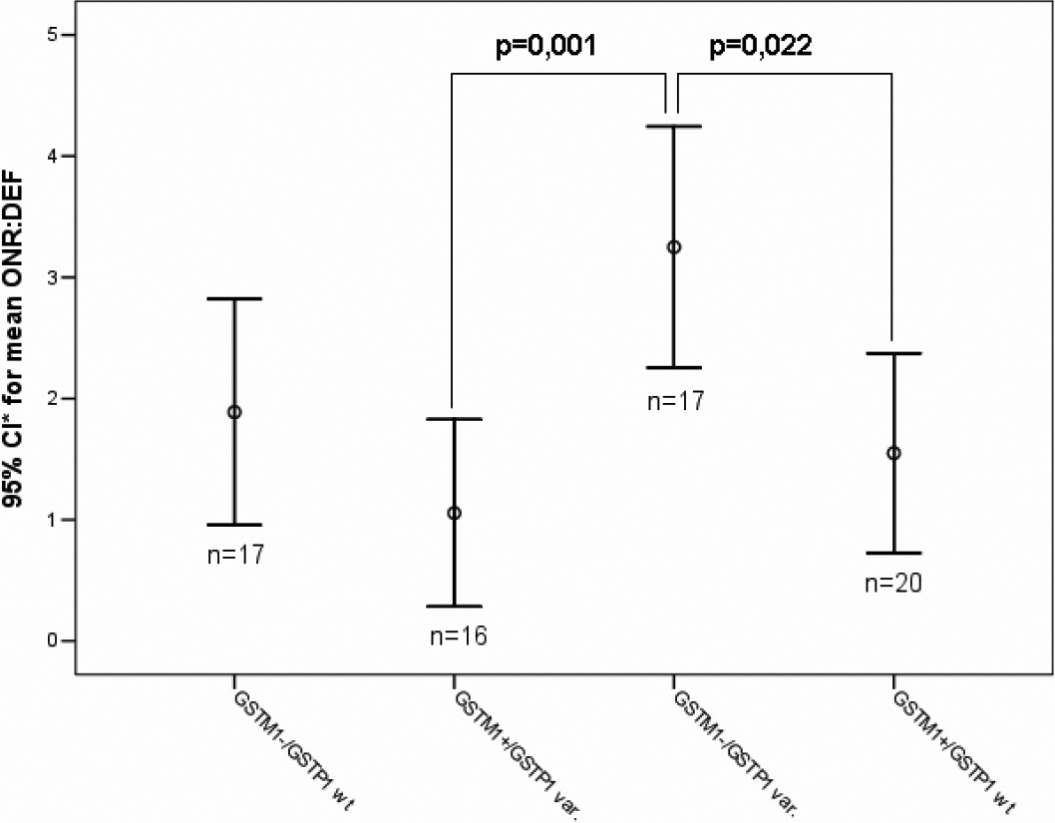
Differences of means for the parameter of optic nerve damage (ONR: DEF) of the right eye for the combined genotypes of GSTM1 and GSTP1. The POAG patients carrying the higher-risk genotype combination of GSTM1 and GSTP1 (GSTM1-/GSTP1 variant) presented higher mean value of ONR:DEF than individuals carrying at least one functional copy of GSTM1. *CI: Confidence Interval.

The gene-gene interaction between *GSTM1* and *GSTT1* genotypes was tested using ANOVA and shows a statistically significant difference for the variable initial IOP of the right eye (F=4,43; p=0.007; Figure 1) and left eye (F=4,32; p=0.008); and optic nerve damage (F=3,32; p=0.024). The interaction of *GSTM1* and *GSTP1* genotypes was statistically significant for initial IOP (F=3,62; p=0.018), visual field defect (3,78; p=0.014) and optic nerve damage (F=5,6; p=0.002; Figure 2).

## Discussion

Although POAG physiopathology still remains partially unclear, there are evidences that reactive oxygen species (ROS) and oxidative stress may play an important role [12,13]. Other study has suggested that oxidative stress may be involved directly in optic nerve neuropathy due to retinal ganglion cells (RGC) damage [14]. Furthermore, ROS may compromise TM integrity favoring IOP increase which is believed to be the major risk factor of POAG [15].

Our results indicate that the oxidative damage may be considered as one of ethiopathogenic factors involved in both POAG development and the more severe phenotypes of this disease. We found that *GSTM1* null genotype, alone, was associated with a more than twofold risk among POAG patients. When combined with the *GSTT1* positive genotype and the variant genotypes of *GSTP1* (dominant model), there was a risk of 2.4 and 2.7 (OR), respectively. The *GSTM1* null genotype was associated with higher IOP levels (p=0.009), more severe damage to the optic nerve (p=0.006) and visual field (p=0.033). We suggest that this genotype may be considered one of the genetic risk factors for the development of POAG and could be used as a genetic marker for the disease. The *GSTT1* null genotype was more frequent in the control group than in the case group. Although this increased prevalence was not statistically significant, but it is suggestive of protection among the carriers. This hypothesis has been suggested by Kim et al. [16] and Rebbeck [17] in association studies involving hepatocellular carcinoma and head and neck cancer, degenerative diseases where oxidative stress was shown to play an important role, like POAG. Additionally, it was shown that workers with *GSTT1* null genotype exposed to polycyclic aromatic hydrocarbons had lower concentration of 8-oxyguanine, the main biomarker of oxidative DNA damage, when compared to individuals with *GSTT1* positive genotype [18].

The association of the *GSTM1* null genotype with POAG has been previously described in other studies [15,19] and was first observed by Izzotti et al. [20]. These authors found a higher risk of POAG development (OR=15) among *GSTM1* null carriers when compared with healthy volunteers. They also observed a more than threefold increase of 8-hydroxy-2´-deoxyguanosine (8-OH-dG) levels, an indicator of oxidative DNA damage in the TM tissue of POAG patients among *GSTM1* null phenotype carriers. *GSTM1* non null carriers did not show an increase of 8-OH-dG levels. The TM tissue was used by the authors to identify *GST* genotypes because TM is believed to be the tissue where primary damage is thought to occur. Their result is considered a new perspective in understanding POAG physiopathology and in elucidating the role of oxidative stress in glaucoma [21]. Yildirim et al. [22] on analyzing a Turkish population, also found higher *GSTM1* null genotype frequencies in POAG patients when compared with healthy volunteers. They described 1.6 higher risk of development of POAG. Contrary to our results, Juronen et al. [6] found that the *GSTM1* positive genotype was associated with higher risk of development of POAG when compared with control group (OR=1.83) while Jansson et al. [8] could not demonstrate any significant association between POAG and GST polymorphisms.

GST enzymes protect cells against electrophilic compounds, endogenous oxidants, and end products formed as secondary metabolites during oxidative stress. Abu-Mero et al. [23] had suggested that decrease GST enzyme activity may contribute to glaucomatous optic neuropathy, although the exact mechanism still remains to be elucidated. It is possible that decreased GST enzyme activity might contribute to oxidative damage directly at retinal ganglion cells in genotype null carriers, or perhaps, any alteration in catalytic activity of the drug-metabolizing enzymes such as GSTM1 may compromise detoxification in TM thus leading to POAG.

In fact, some studies have demonstrated that chronic oxidative stress may compromise the integrity of the TM [24,25]. Green et al. [25] demonstrated that TM cells submitted to long period of exposure to H_2_O_2_ in aqueous humor, had altered defense mechanisms and lowered their cell numbers in the in vitro studies. Although catalases catalyze degradation of H_2_O_2_ at higher concentrations, there are evidences that GST protects ocular tissues against damage when lower concentrations of H_2_O_2_ are present [19]. Repeated oxidative stress events may compromise adherence of TM cells leading to out flow of the aqueous humor [26]. In POAG patients, it is plausible to say that chronic oxidative stress related to aging, an established risk factor for the development of glaucoma, may be an additional risk factor for progression to glaucomatous disease.

In our study, the right eye showed more relevant alterations in higher IOP levels, visual fields and optic nerve defects. These results are in agreement with the observation of asymmetry between both eyes in glaucomatous disease observed in clinical practice. It also suggest that besides genetic factors, there are other factors are implicated in this complex disease [27].

In summary, our study is the first case-control to try to associate *GST* genotype and POAG in the American continent combined with clinical evaluations like IOP, optic nerve, and visual field defects. The population under study is located in an area supposedly composed of genetic diversity established over 500 years, which consists of European immigrants (mainly Portuguese), Africans and the Native American population (Tupinamba). Moreover, it is the first study to identify an increased frequency of association of *GSTM1* null genotype and *GSTP1*Ile/Val or *GSTP1*Val/Val in patients with glaucoma and the influence of these polymorphisms with severity of the disease. We would like to note here, that analysis of other enzyme polymorphisms is necessary to be undertaken to further elucidate the pathophysiology of this complex disease. It might be possible in the near future to perform genetic based diagnoses in the early stages of the disease, thus preventing blindness associated with the disease.

An important limitation of the present study is the small sample size, which leads to considerable lack of statistical power. Thus, the results should be considered with caution. Although this study has achieved a near to or higher than 80% power to detect several statistically significant results (Appendix 1), but for the association between *GSTM1* and POAG, the observed power was only 66%. Therefore, further studies are suggested to be done in other populations and with higher sample sizes, to confirm the influence of *GST*s polymorphisms in the pathophysiology of primary open angle glaucoma.

## Appendix 1.

Power analysis of the positive findings reported in present study. To access the data, click or select the words “Appendix 1.” This will initiate the download of a compressed (pdf) archive that contains the file.
